# Metal–Organic Frameworks (MOF)-Derived Gel Electrolyte via UV Cross-Linking for High-Performance Lithium Metal Batteries

**DOI:** 10.3390/gels11060409

**Published:** 2025-05-29

**Authors:** Naiyao Mao, Lingxiao Lan, Qiankun Hun, Jianghua Wei, Xinghua Liang, Yifeng Guo

**Affiliations:** 1 Guangxi Key Laboratory of Automobile Components and Vehicle Technology, Guangxi University of Science & Technology, Liuzhou 545006, China; maonaiyao@gmail.com (N.M.); llx2685062@163.com (L.L.); 13383371921@163.com (Q.H.); 2 School of Mechanical Engineering, Chengdu University, Chengdu 610106, China; guobujia2000@163.com

**Keywords:** gel electrolyte, Metal–Organic Frameworks, PVDF-HFP, UV curing technology, lithium metal batteries

## Abstract

Gel electrolytes (GEs) play a pivotal role in the advancement of lithium metal batteries by offering high energy density and enhanced rate capability. Nevertheless, their real-world application is hampered by relatively low ionic conductivity and significant interfacial resistance at room temperatures. In this work, we developed a gel electrolyte membrane (GEM) by embedding Zeolitic Imidazolate Framework-8 (ZIF-8) metal–organic frameworks (MOFs) material into a poly(vinylidene fluoride-co-hexafluoropropylene) (PVDF-HFP) matrix through UV curing. The composite membrane, with 4 wt% ZIF-8, exhibited an ionic conductivity of 1.17 × 10^−3^ S/cm, an electrochemical stability window of 4.7 V, and a lithium-ion transference number of 0.7. The test results indicate that the electrochemical performance of LFP//GEM//Li battery has an initial specific capacity of 168 mAh g^−1^ at 0.1 C rate. At 1 C, the discharge capacity was 88 mAh g^−1^, and at 2 C, it was 68 mAh g^−1^. Enhanced ionic transport, improved electrochemical stability, and optimized lithium-ion migration collectively contributed to superior rate performance and prolonged cycle life. This study offers novel insights and methodological advances for next-generation lithium metal batteries technologies.

## 1. Introduction

Recent advancements in electric vehicle (EV) technology and energy storage infrastructure for power grids have driven the need for improved electrochemical performance in lithium metal batteries (LMBs). At the same time, market demands have become increasingly rigorous, particularly regarding energy density and safety, which are now key considerations in modern battery design [1,2]. Among the emerging solutions, LMBs utilizing gel electrolytes have attracted significant attention due to their potential to surpass the energy density limits of conventional liquid electrolyte systems [3]. However, several challenges persist. These include high interfacial energy barriers that impede lithium-ion transport [4], poor rate performance, and limited cycle life. Additionally, gel electrolytes typically exhibit lower ionic conductivity at room temperature compared to their liquid counterparts [5,6]. Addressing these limitations, the development of advanced gel electrolyte membranes that offer both high ionic conductivity and stable interfaces has become a critical focus in the field of electrochemical energy storage [7].

The application of polymer electrolyte materials in lithium metal batteries has made significant progress [8,9,10]. Poly(vinylidene fluoride) (PVDF)-based polymers, as high molecular weight materials, have been widely employed in the early development of gel electrolyte membranes due to their highly stable chemical properties and outstanding mechanical durability [11,12]. In recent years, researchers have synthesized PVDF-HFP copolymers by copolymerizing vinylidene fluoride (PVDF) with hexafluoropropylene (HFP) [13], which increased the proportion of amorphous regions in the copolymer, markedly enhancing ion transport properties [14,15]. Liang et al. further improved the system by incorporating garnet-type Li_7_La_3_Zr_2_O_12_ (LLZO) nanoparticles into the PVDF-HFP matrix. The plasticizing effect of LLZO particles endowed the composite with excellent electrochemical performance and cycling stability [16]. Sohn et al. fabricated porous polyethylene separators coated with a PVDF-HFP/PMMA composite via a simple dipping process under 40% relative humidity, achieving a maximum ionic conductivity of 1.69 mS/cm [17].

Metal–organic frameworks (MOFs) are a class of porous materials with tunable pore sizes and exceptionally high specific surface areas, offering a novel approach to the design of lithium metal batteries [18,19]. These materials feature well-ordered channel structures and high porosity [20], which provide continuous pathways and abundant active sites for ion transport. Moreover, the organic ligands and metal nodes in MOFs can be flexibly modified through chemical functionalization to optimize their electrochemical performance [21]. The high thermal stability of MOFs [22], along with their composite structures with polymers, enhances the mechanical strength of electrolytes, suppresses lithium dendrite growth, and accommodates volume changes during charge–discharge cycles [23]. Wang et al. constructed a three-dimensional MOF crosslinked network asymmetric solid polymer electrolyte (SPE) via in situ polymerization. By selectively sieving anions through the MOF layers, the lithium-ion transference number was elevated to 0.72. The nanostructured MOF layers serve as a shield, effectively suppressing Li dendrite growth and ensuring uniform Li^+^ transport [24]. Sun et al. proposed a novel design for a polyethylene oxide (PEO)-based SPE by incorporating activated Copper 1,3,5-phenyltricarboxylic acid (HKUST-1) MOFs into the PEO electrolyte. This approach not only improved the lithium transference number and mechanical properties but also enhanced the overall electrochemical performance [25]. Dong et al. combined experimental and theoretical methods to systematically study the effects of pore size and metal vacancies in porous metal oxides (MOFs) on ion transport characteristics and electrochemical stability. It demonstrated that a metal–organic framework electrolyte comprising non-electroactive metal coordination nodes demonstrates at least 20% wider voltage tolerance range relative to redox-active metal node analogues [26].

UV crosslinking offers notable advantages in the fabrication of battery electrolyte membranes. Unlike conventional thermal crosslinking methods, UV crosslinking does not require high temperatures. This reduces the risk of thermal damage to the membrane and helps maintain the uniformity of its internal structure. Furthermore, UV crosslinking significantly enhances production efficiency, as it typically requires much shorter reaction times compared to thermal methods [27]. For example, Kim et al. employed UV photopolymerization to synthesize a cross-linked polymer matrix composed of poly(ethylene glycol) diacrylate (PEGDA) and dipentaerythritol hexaacrylate (DPHA). When applied in lithium-ion batteries, the resulting membrane achieved a high lithium-ion transference number of 0.65 [28].

In this study, a rigid flexible strategy is used for the design of gel electrolytes. The metal–organic framework (MOF) is used as a support to realize the high mechanical strength of the electrolytes. The flexible polymer PVDF-HFP enables the electrolyte to adapt to the volume change in the internal materials of the battery during service. PVDF-HFP and Zeolitic Imidazolate Framework-8 (ZIF-8) were utilized and processed via a rapid UV-curing technique to fabricate the poly(vinylidene fluoride-co-hexafluoropropylene)/ZIF-8 composite (abbreviated as PHZ) with excellent performance. The incorporation of ZIF-8 enabled the formation of ion channels and modulated the mobility of polymer chains, synergistically enhancing Li^+^ transport. Consequently, this method promotes the advancement of lithium metal batteries with excellent electrochemical performance and enhanced safety.

## 2. Results and Discussion

**Figure 1:**
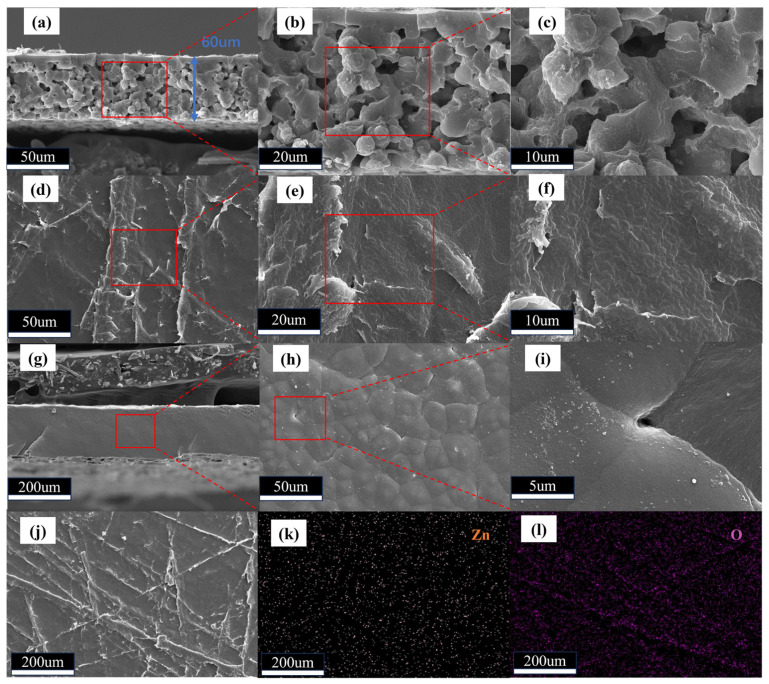
(**a**–**c**) Sectional view SEM images of PHZ electrolyte membranes. (**d**–**f**) Surface SEM images of PHZ electrolyte membranes. (**g**–**i**) Cross-sectional and surface SEM images of PVDF-HFP electrolyte membranes. (**j**–**l**) Elemental distribution of Zn and O in PHZ electrolyte membranes.

As shown in Figure 1a–c,g–i, by analyzing the cross-sectional scanning electron microscopy (SEM) images of the electrolyte membranes, it is evident that, compared to the PVDF-HFP electrolyte membrane without MOFs, the PVDF-HFP matrix with added ZIF-8 forms a well-connected porous network structure. In the subsequent conductivity test, the conductivity of PHZ film was one unit higher than that of PVDF-HFP film. It indicates that the incorporation of MOFs into PVDF-HFP facilitates the construction of effective ion transport channels, thereby enhancing lithium-ion conduction [29].

Meanwhile, in contrast to membranes without MOFs, the surface SEM images of PHZ electrolyte membranes exhibit a smooth morphology and seamless integration between ZIF-8 and PVDF-HFP, demonstrating strong mutual integration between the two components. Furthermore, as shown in Figure 1j,k,l the uniform distribution of Zn elements further confirms that the adopted preparation method ensures a homogeneous dispersion of ZIF-8 within the composite material.

**Figure 2:**
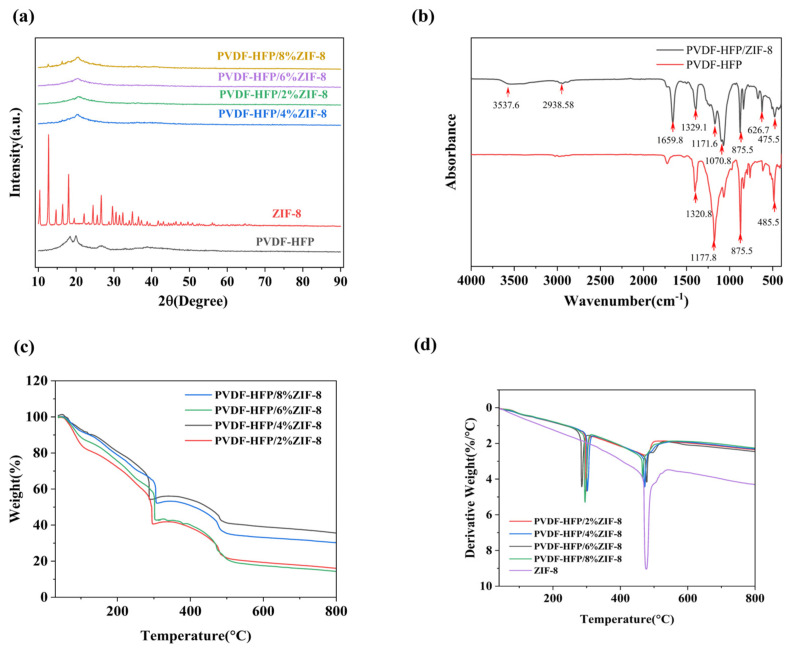
(**a**) XRD patterns of ZIF-8, PVDF-HFP, and their composite. (**b**) FTIR spectra of PHZ and its synthetic materials. (**c**) Thermogravimetric (TG) curves of PHZ materials with different proportions of ZIF-8 added. (**d**) Differential Scanning Calorimeter (DSC) curves of PHZ materials with different ratios of ZIF-8 added.

The XRD patterns of the PHZ electrolyte membrane are shown in Figure 2a. The XRD spectrum of PVDF-HFP primarily exhibits a broad peak around 2θ ≈ 18° to 20°, indicating its low crystallinity and high amorphous content [30]. After the incorporation of ZIF-8, the peak disappears and the amorphous region increases, suggesting that ZIF-8 hinders the orderly arrangement of PVDF-HFP molecular chains, suppressing nucleation and crystal growth, ultimately leading to a decrease in crystallinity [31]. A higher proportion of the amorphous region reduces the entanglement of molecular chains and increases the inter-chain voids (free volume), providing space for segmental motion and Li^+^ migration, thereby enhancing lithium-ion transport [32].

In order to further investigate the interaction between polymers and MOFs, FTIR spectroscopy analysis was performed on PHZ electrolyte membranes and PVDF-HFP. The FTIR spectrum provides the following insights: the peak at 3537 cm^−1^ corresponds to the coordination of the C-O-C bond, which is typically attributed to the interaction between Li^+^ and ether oxygen. The peaks at 2938 cm^−1^ (C–H stretching vibration), 1329 cm^−1^ (C-F stretching vibration), and 626 cm^−1^ (CF_2_ out-of-plane bending vibration) confirm the presence of the PVDF-HFP backbone. The peak at 875 cm^−1^ suggests that the HFP copolymer effectively reduces crystallinity. The peak at 1659 cm^−1^ corresponds to the C=O stretching vibration in PVDF. Additionally, the peak at 475 cm^−1^ is attributed to the vibration between the metal and organic ligand, representing the Zn–N stretching vibration in ZIF-8, confirming its successful incorporation into the polymer matrix. The peaks at 1171 cm^−1^ (C–F stretching vibration) and 1070 cm^−1^ indicate the presence of ClO_4_^−^ in the polymer [33].

Thermogravimetric analysis (TGA) reveals that below 300 °C, the composite material undergoes mass loss due to the desorption of adsorbed water, residual solvents, or low-molecular-weight volatile components, as well as the decomposition of lithium salt. The intermediate stable stage (350–450 °C) indicates that the main structure (PVDF-HFP crystalline region and ZIF-8) maintains good thermal stability within this temperature range. As the temperature increases, the PVDF-HFP polymer backbone undergoes substantial breakdown, with its typical degradation process accompanied by the release of gases such as HF, leading to a sharp mass loss. Simultaneously, the organic ligands in ZIF-8 also enter their decomposition phase, further accelerating the overall weight reduction. The addition of lithium salt causes the decomposition temperature of ZIF-8 to shift earlier [34]. After decomposition, ZIF-8 may form inorganic products such as ZnO, which are relatively stable [35]. DSC testing also showed that PHZ material exhibited significant decomposition reactions at 300 °C, accompanied by endothermic peaks, corresponding to the weight loss changes in PHZ at different ratios in TG testing. A clear endothermic peak also appeared at around 500 °C, corresponding to the change in TG weight loss of ZIF-8 in PHZmaterial.

**Figure 3:**
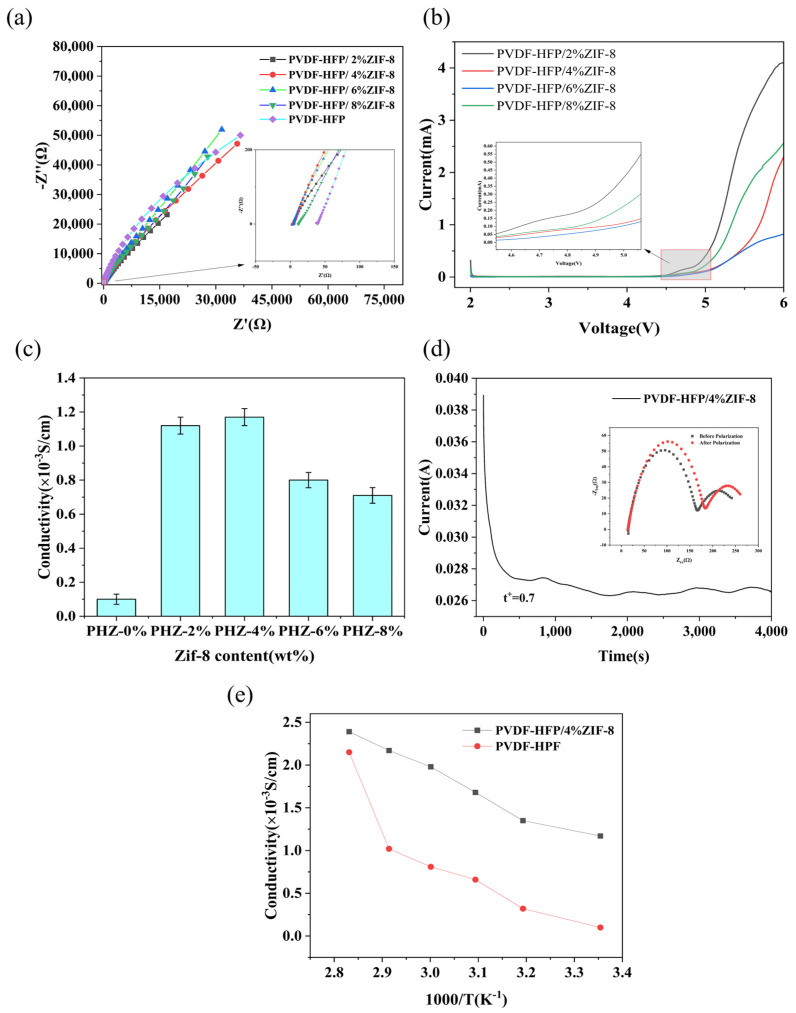
(**a**) Conductivity diagram of PHZ composite electrolyte membranes with different mass fractions of ZIF-8. (**b**) Electrochemical window test of PHZ electrolyte membranes. (**c**) Bar chart comparing conductivity with different mass fractions of ZIF-8. (**d**) Test chart of ion migration number of PHZ electrolyte membranes. (**e**) Comparison of conductivity between PHZ electrolyte membranes and PVDF-HFP electrolyte membrane at different temperatures.

Polymer gel electrolyte membranes with different proportions of ZIF-8 were systematically tested using coin-type symmetric cells and an electrochemical workstation. As shown in Figure 3c, the results indicate that when 4% ZIF-8 is added to the electrolyte membrane, its ionic conductivity reaches the highest value of 1.17 × 10^−3^ S/cm. However, with a further increase in ZIF-8 content, the ionic conductivity gradually decreases. This phenomenon can be attributed to the intensified trends of particle agglomeration and interfacial blocking effects when the ZIF-8 loading surpasses 4%, resulting in the obstruction of ion transport pathways and subsequent impairment of ionic transport kinetics. Compared to pure PVDF-HFP electrolyte membranes (1 × 10^−4^ S/cm), the conductivity of PHZ increases by an order of magnitude.

Additionally, Figure 3b shows the linear sweep voltammetry (LSV) curves of PHZ electrolyte membranes with different ZIF-8 addition ratios. When the amount of ZIF added is 4%, the electrochemical window of the electrolyte membrane expands to 4.7 V, which is significantly better than that of PHZ membranes with other addition ratios. In PVDF-HFP-based electrolyte membranes, excessive addition of MOF may lead to insufficient binder and affect the mechanical strength of the membrane. If it is too low, it cannot effectively regulate the pore structure. Therefore, an appropriate ratio of MOF to PVDF HFP can expand the range of battery LSV [36]. The porous network structure of ZIF-8 forms additional ion-conduction pathways within the polymer matrix, thereby accelerating ion migration. Given that the PHZ electrolyte membrane achieved the highest conductivity when 4% of zif-8 was added, we conducted lithium migration tests on this specific proportion of PHZ, as illustrated in Figure 3d. The synergistic effect between ZIF-8 and the local segmental motion of the amorphous region in PVDF-HFP enhances lithium-ion transference [37,38], achieving a lithium-ion transference number (TLi^+^) of 0.7.

**Figure 4:**
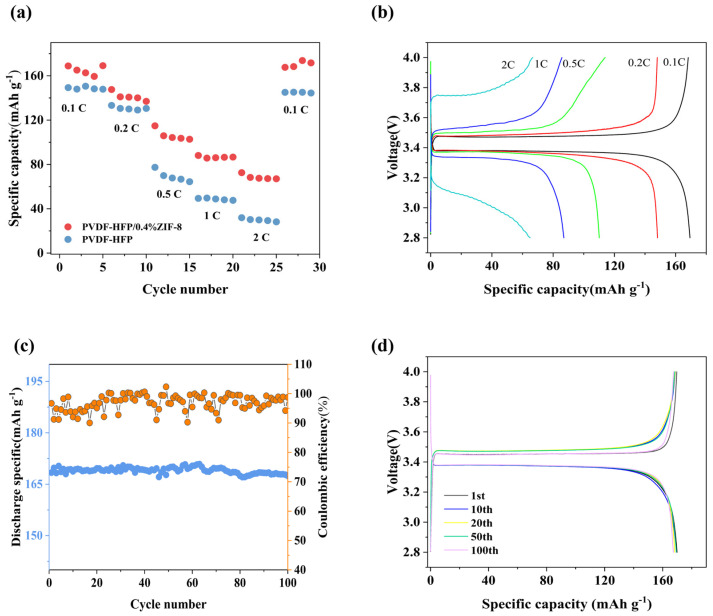
(**a**) Rate performance of PVDF-HFP and PHZ batteries at 25 °C. (**b**) Charge–discharge curves of the PHZ battery at 0.1 C, 0.2 C, 0.5 C, 1 C, and 2 C. (**c**) Cycling performance of the PHZ battery over 100 cycles at 25 °C. (**d**) Charge–discharge curves of the PHZ battery over 100 cycles.

To further evaluate the electrochemical performance of the PHZ solid-state battery, rate performance and cycling tests were conducted at room temperature (25 °C) by an electrochemical workstation (Figure 4a, b). The initial discharge capacity is 168 mAh g^−1^. At a discharge rate of 0.2 C, the discharge capacity was 141 mAh g^−1^, while at 0.5 C, it was 105 mAh g^−1^. At 1 C, the discharge capacity was 88 mAh g^−1^, and it was 68 mAh g^−1^ at 2 C. When the discharge rate returned to 0.1 C, the battery still maintained a discharge capacity of 168 mAh g^−1^. In contrast, the solid-state battery with the PVDF-HFP electrolyte membrane (without ZIF-8) exhibited an initial discharge capacity of only 149 mAh g^−1^ at 0.1 C. At 0.2 C, the discharge capacity was 133 mAh g^−1^; at 0.5 C, it was 77 mAh g^−1^; at 1 C, it was 48 mAh g^−1^; and at 2 C, it was 28 mAh g^−1^. Comparatively, the porous structure of PHZ provides more space for lithium-ion insertion and storage, allowing more lithium ions to participate in the reaction under the same active material content. This enhances the discharge-specific capacity and enables efficient ion transport channels, leading to excellent high-rate performance. The nano confinement effect of MOFs can suppress the possible chain breakage or crystallization of PVDF-HFP in long-term cycling, thereby promoting the integrity and stability of PHZ structures [39].

In the cycling test, the PHZ solid-state battery exhibited an initial discharge capacity of 169 mAh g^−1^ at a 0.1 C discharge rate. After 100 cycles, the remaining discharge capacity was 162 mAh g^−1^, with a capacity retention rate of 95.8% and a Coulombic efficiency of 99.5% (Figure 4c). The polarization remained around 100 mV(Figure 4d), with negligible change after cycling, demonstrating the outstanding cycling stability of PHZ.

## 3. Conclusions

In summary, we successfully fabricated the PHZ polymer electrolyte membrane by incorporating the metal–organic framework material ZIF-8 into PVDF-HFP and employing UV light-curing rapid prototyping for full polymerization. The porous network structure of ZIF-8 formed additional ion transport channels within the polymer matrix, accelerating lithium-ion migration. The uniform Li^+^ flux distribution regulated by ZIF-8 channels effectively reduced dendrite growth risks and improved battery cycling stability. The PHZ polymer electrolyte membrane with 4 wt% ZIF-8 exhibited an ionic conductivity of 1.17 × 10^−3^ S/cm, an electrochemical stability window of 4.7 V, and a lithium transference number of 0.7. The solid-state battery assembled with PHZ polymer electrolyte demonstrated an initial discharge capacity of 168 mAh g^−1^ at a 0.1 C discharge rate, with a capacity retention rate of 95.8% and a Coulombic efficiency of 99.5% after 100 cycles. at 1 C, it was 48 mAh g^−1^; and at 2 C, it was 28 mAh g^−1^. These results indicate excellent rate and cycling performance. Therefore, the PHZ electrolyte material offers a novel approach and method for future lithium metal batteries research and development.

## 4. Materials and Methods

### 4.1. Materials

The raw materials include lithium perchlorate (LiClO_4_, 99%, Aladdin, Shanghai, China), poly(vinylidene fluoride-co-hexafluoropropylene) (PVDF-HFP, Mw = 600,000, Macklin, Shanghai, China), N,N-dimethylformamide (DMF, 99.8%, Aladdin, Shanghai, China), polyvinylidene fluoride (PVDF, Arkema, France); 1-methyl-2-pyrrolidone (NMP, Aladdin, Shanghai, China); conductive carbon black (99.9%, Kejing, Shenzhen, China); lithium iron phosphate (LiFePO_4_, LFP, 99.9%, Macklin, Shanghai, China); zeolitic imidazolate framework-8 (ZIF-8, 99%, Tanyu New Materials, Guangdong, China); 2-hydroxy-2-methylpropiophenone (1173, Chembridge, Beijing, China); polyurethane acrylate (PUA, RYOJI, Frankfurt, Germany); ethoxylated trimethylolpropane triacrylate (ETPTA, Mn = 428, Macklin, Shanghai, China).

### 4.2. Preparation of Electrolyte Membrane

**Figure 5:**
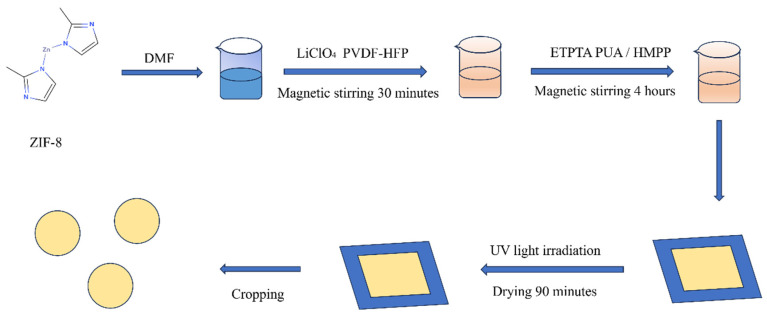
Preparation process of PHZ polymer.

A total of 8 g of N,N-dimethylformamide (DMF) was measured into a beaker, and the zeolitic imidazolate framework-8 (ZIF-8) MOFs were dispersed into the DMF. The mixture was sonicated for 30 min to ensure complete dispersion. Subsequently, poly(vinylidene fluoride-co-hexafluoropropylene) (PVDF-HFP) and a specific amount of lithium perchlorate (LiClO_4_) were dissolved/dispersed into the ZIF-8/DMF solution. The solution was stirred using a magnetic stirrer at 45 °C with a stirring speed of 450 r/min for 4 h to ensure thorough mixing. After stirring, ethoxylated trimethylolpropane triacrylate (ETPTA) and polyurethane acrylate (PUA) were added to the solution, followed by an additional 15 min of stirring. Finally, 2-hydroxy-2-methylpropiophenone (HMPP) was introduced, and the solution was stirred for another 15 min. The resulting mixture was transferred to an ultrasonic device and sonicated for 30 min to remove air bubbles. The prepared polymer composite consisted of PVDF-HFP and ZIF-8, with the mass fraction of ZIF-8 in the composite set at 0 wt%, 2 wt%, 4 wt%, 6 wt%, and 8 wt%. These samples are hereafter referred to as PHZ-0%, PHZ-2%, PHZ-4%, PHZ-6%, and PHZ-8%, respectively. The production process is shown in Figure 5.

The prepared material was poured onto a polytetrafluoroethylene (PTFE) plate and spread into a thin film using a scraper. The film was then placed in a UV curing machine for photopolymerization and subsequently vacuum-dried at 60 °C for 90 min (The light source is an ultraviolet lamp, with a peak wavelength of 365 nm). Finally, the electrolyte membrane was peeled off and cut into small circular discs with a diameter of 19 mm. The discs were stored in a glovebox (<0.01 PPM H_2_O and O_2_) for future use.

### 4.3. Battery Assembly

The lithium metal battery was assembled using an electrolyte membrane, a LiFePO_4_ (LFP) cathode, and a lithium (Li) anode. The cathode was fabricated by coating an aluminum foil with a slurry mixture containing 80 wt% lithium iron phosphate (LFP), 10 wt% polyvinylidene fluoride (PVDF) binder, and 10 wt% carbon black additive. This cathode configuration achieved an active material mass loading of about 1.8 mg cm^−2^. After cell assembly, the battery was stored in an argon-filled glovebox for 12 h, followed by a 1 h drying process in a vacuum oven maintained at 60 °C.

### 4.4. Material Characterization

The thermal decomposition curve was obtained using a thermogravimetric (TG) analyzer (Manufacturer: METTLER TOLEDO, Greifensee, Switzerland, Model: DSC 3+). For this analysis, PHZ samples were weighed (mg) and subjected to thermal analysis in the temperature range of 30–800 °C at a heating rate of 10 °C/min. The thermal analysis was conducted under a nitrogen (N_2_) atmosphere with a flow rate of 100 mL/min. Fourier-transform infrared (FTIR) spectra were recorded using an FTIR spectrometer (Manufacturer: Thermo, Waltham, MA, USA, Model: Nicolet iS5) operating in attenuated total reflectance (ATR) mode. Infrared measurements were performed by mixing a small amount of potassium bromide (KBr) to absorb polymer peaks in the wavenumber range of 4000–400 cm^−1^, with a resolution of 4 cm^−1^. Phase analysis of the materials was conducted using an X-ray diffractometer (Manufacturer: Dandong Haoyuan, Dandong, China, Model: DX-2700, Cu-Kα, 40 kV × 30 mA). The scanning range was set from 5° to 80° (2θ), with a scanning rate of 0.5° and a step size of 0.1° min^−1^. The overall morphology of the composite materials was observed using a scanning electron microscope (SEM) (Manufacturer: Hitachi, Tokyo, Japan, Model: HITACHI SU8010). The cross-section of the materials was obtained via liquid nitrogen freeze-fracture to examine the internal pore distribution and the embedding state of ZIF-8 particles.

### 4.5. Electrochemical Testing

Ionic conductivity can reflect whether polymer gel electrolytes can meet the standards of solid-state batteries. A coin cell symmetric configuration (stainless steel (SS)/CSE/SS) was assembled and tested on an electrochemical workstation (DH 7000, Jiangsu, China) using electrochemical impedance spectroscopy (EIS) in the frequency range of 0.1 Hz to 1 MHz with an amplitude of 10 mV. The ionic conductivity (σ) can be calculated using the following equation:

(1) $$\sigma =\frac{L}{RS}.$$

In this equation, *L* denotes the electrolyte membrane thickness (cm), *R* is the bulk resistance (Ω) obtained from EIS fitting, and *S* represents the effective contact area (cm^2^) between the electrode and the electrolyte membrane.

The lithium-ion transference number (*t_Li_^+^*) is a key parameter for evaluating the contribution of lithium ions to the total ionic conductivity of the electrolyte and serves as an important criterion for determining the performance of lithium-ion batteries. The *t_Li_^+^* of the composite gel electrolyte (CGE) is measured using a symmetric cell (Li/CGE/Li) by combining electrochemical impedance spectroscopy (EIS) and direct current (DC) polarization on an electrochemical workstation. First, the initial impedance value (*R*_0_) is obtained through EIS testing. A small DC voltage is then applied, and the current is allowed to reach a steady-state value (*Is*). After removing the DC voltage, the impedance spectrum is measured again to obtain the interfacial resistance after polarization (*R_s_*).

The lithium-ion transference number (*t_Li_^+^*) is calculated using the following equation:

(2) $$t_{{Li}^{+}}=\frac{I_{s}(\Delta V-R_{0}I_{0})}{I_{0}(\Delta V-R_{S}I_{S})}.$$

In Equation (2), *R*_0_ and *R_s_* represent the impedance values before and after DC polarization, respectively; Δ*V* is the applied voltage across the battery (10 mV); and *I*_0_ and *I_s_* denote the initial and steady-state currents, respectively [40].

To assess the electrochemical stability range of the CGE, a Li/CGE/WE cell was built and subjected to linear sweep voltammetry (LSV) over a voltage span of 2.0–6.0 V (vs. Li/Li^+^) at a scan rate of 0.5 mV/s.

The rate performance and cycling stability of the battery were evaluated using a battery testing system (Neware, Dongguan, China). Coin cells were assembled with LiFePO_4_ as the cathode and lithium metal as the anode, and their cycling and rate performance were measured. The tests were conducted within a voltage range of 2.8–4.0 V.

## Data Availability

The data are contained within the article.
